# Fast Detection of Heat Accumulation in Powder Bed Fusion Using Computationally Efficient Thermal Models

**DOI:** 10.3390/ma13204576

**Published:** 2020-10-14

**Authors:** Rajit Ranjan, Can Ayas, Matthijs Langelaar, Fred van Keulen

**Affiliations:** Maritime and Materials Engineering, Department of Precision and Microsystems Engineering (PME), Faculty of Mechanical, Delft University of Technology, 2628CD Delft, The Netherlands; C.Ayas@tudelft.nl (C.A.); M.Langelaar@tudelft.nl (M.L.); A.vanKeulen@tudelft.nl (F.v.K.)

**Keywords:** additive manufacturing, laser powder bed fusion, heat transfer process modeling, physics-based simplifications

## Abstract

The powder bed fusion (PBF) process is a type of Additive Manufacturing (AM) technique which enables fabrication of highly complex geometries with unprecedented design freedom. However, PBF still suffers from manufacturing constraints which, if overlooked, can cause various types of defects in the final part. One such constraint is the local accumulation of heat which leads to surface defects such as melt ball and dross formation. Moreover, slow cooling rates due to local heat accumulation can adversely affect resulting microstructures. In this paper, first a layer-by-layer PBF thermal process model, well established in the literature, is used to predict zones of local heat accumulation in a given part geometry. However, due to the transient nature of the analysis and the continuously growing domain size, the associated computational cost is high which prohibits part-scale applications. Therefore, to reduce the overall computational burden, various simplifications and their associated effects on the accuracy of detecting overheating are analyzed. In this context, three novel physics-based simplifications are introduced motivated by the analytical solution of the one-dimensional heat equation. It is shown that these novel simplifications provide unprecedented computational benefits while still allowing correct prediction of the zones of heat accumulation. The most far-reaching simplification uses the steady-state thermal response of the part for predicting its heat accumulation behavior with a speedup of 600 times as compared to a conventional analysis. The proposed simplified thermal models are capable of fast detection of problematic part features. This allows for quick design evaluations and opens up the possibility of integrating simplified models with design optimization algorithms.

## 1. Introduction

Additive manufacturing (AM) offers unprecedented design freedom as compared to conventional manufacturing techniques. The layer-by-layer material deposition allows for manufacturing functional parts with high geometric complexity. Due to this advantage, AM has already gained significant popularity among manufacturing industries such as automotive, aerospace and medical [1,2]. However, the AM process is not free from manufacturing constraints and, if overlooked, these constraints can cause a wide range of defects in the final part which lead to an increased overall cost. Therefore, to fully capitalize on the benefits offered by AM, manufacturing limitations should be considered at the design stage.

Laser powder bed fusion (LPBF) process is one of the most common techniques for printing metal parts. It involves sequential deposition of metal powder layers which are selectively molten and fused together in predefined areas using a moving laser beam. This implies that heat primarily flows from each newly deposited topmost layer towards the thick baseplate at the bottom, which acts as a heat sink. It is observed that whenever the deposited heat is not properly evacuated towards the baseplate, it leads to local overheating or heat accumulation [3]. It typically refers to a situation where material locally experiences thermal process conditions that result in temperatures outside the desired temperature range needed to obtain the desired final product quality. Given the wide range of potential temperature-induced production failure mechanisms, overheating can manifest itself in many forms. In the in situ monitoring studies conducted by Hooper [4] and Kruth [5], overheating is characterized by the enlarged melt pool observed in the vicinity of lower conductivity regions which obstruct heat flow. In the numerical study conducted by Hodge et al. [6], the overheating phenomenon is characterized by the overshoot of simulation temperatures above the melting point.

It has been widely recognized in the literature that local overheating or heat accumulation is detrimental for final part quality and can cause various defects. For example, Mertens et al. [3] investigated the defect of dross formation where an enlarged melt pool caused by local overheating leads to undesired sintering of loose powder in its vicinity. Charles et al. [7] also studied dross formation by investigating the correlation between process parameters, e.g., laser power, scan speed and the resulting surface roughness. High process temperatures along with the effect of surface tension lead to the defect of melt ball formation [8]. These cases are examples where overheating depends on the local distribution of conductivity in the vicinity of melt pool. Another more recently studied phenomenon is that of gradual accumulation of heat with increasing part height. As more layers are deposited, they act as a thermal barrier causing prolonged heated zones with slower cooling. This becomes increasingly significant in tall parts with high heat capacity. Therefore, the phenomenon was initially investigated for processes such as laser metal deposition (LMD) [9] and wire-arc-based AM process (WAAM) [10], where typical part sizes are larger than LPBF. More recently, it has been studied for the LPBF process as well. For example Hilgenberg [11] used in situ thermography for observing cooling conditions during the process. It is reported that heat accumulation increases with increasing build height. Also, inter-layer time (ILT), i.e., the time elapsed between processing successive layers, is found to be an important parameter which inversely affects heat accumulation.

It is important to understand that both these phenomena, i.e., local overheating and gradual heat accumulation during the build, are intimately coupled. Elevated temperatures due to local overheating contribute to the gradual accumulation in the successive layers and accumulated heat will conversely intensify local overheating. Therefore, both these phenomena jointly determine the overall thermal history of the part, which subsequently has a direct effect on the final part quality. For example, Hilgenberg [11] performed metallographic analysis to study the influence of heat accumulation on observed microstructures. It is suggested in this paper that specific temperature ranges which are important from the context of phase transformations should be considered while analyzing the influence of heat accumulation. Another aspect investigated by Wildman [12] shows that regions susceptible to overheating generate significant residual stresses, which are likely to result in undesired deformations and potentially build failures. Kastsian and Reznik [13] analyzed a part geometry where local overheating led to excessive deformations causing recoater jamming. Heigel and Whitenton [14] presented layer-wise cooling rate maps which show that overheating zones tend to cool slower. Lastly, as suggested by Charles et al. [7], another aspect of overheating control is associated with selecting optimal process parameters. For example, Mertens et al. [3] varied laser power in accordance with the level of overhang for controlling dross formation. However, such approaches also rely on prior knowledge of overheating zones so that parameters can be tuned accordingly. Hence, early identification of geometric features which are prone to local overheating can assist designers and machine operators in judging manufacturability of the final design.

Typically, downfacing or overhanging features are prone to this phenomenon as loose powder, with significantly lower thermal conductivity than that of the bulk material, does not allow for rapid heat evacuation. The degree of overheating typically increases with decreasing the overhang angle defined as the angle between part surface and the base plate. Therefore, heuristic AM design guidelines are used, where features with overhang angles smaller than a critical value are avoided [15,16]. Although overhangs are a salient example of features which cause overheating, the phenomenon is not uniquely linked to them. For example, LPBF specimens manufactured by Zimm. [17] and Patel et al. [18] demonstrate overheating induced discoloration and high surface roughness, respectively, even after strictly following the overhang design guideline. This indicates that not only the local overhang angle, but the thermal response should be considered for detecting and preventing overheating.

Numerical modeling of the transient heat transfer phenomena during the LPBF process can provide thermal history information which can be used for identification of features causing local overheating. However, excessive computational cost associated with detailed modeling of the LPBF process prohibits part-scale implementation, particularly in iterative design settings. Please note that the size of the laser spot is in the order of a few microns whereas the part size is in the order of centimeters. Moreover, in time domain, the phenomenon of melting and re-solidification is extremely fast, whereas cooling between the layers is in order of tens of seconds. Hence, to capture the steep thermal gradients caused by a fast-moving laser, very fine spatial and temporal resolution is required which makes the modeling process computationally intractable. To address this issue, researchers reduce the computational complexity of models by introducing simplifications in various ways. For example, several studies motivate consideration of conduction as the only mode of heat transfer while neglecting convection and radiation [19,20,21]. Another commonly used simplification is to simulate an entire layer addition as a single simulation step, i.e., the entire layer area is simultaneously exposed to a heat flux. It has been demonstrated by Rubenchik [22] that during a new layer deposition, 4–8 previously deposited layers are also re-molten and fused. This re-melting is intentional as it allows for a seamless connection between the layers. Therefore, the concept of “superlayers” or layer lumping has been proposed where deposition of one superlayer refers to the simultaneous deposition of multiple real layers while modeling the process [23,24,25,26].

These simplifications can provide significant computational gains as compared to high-fidelity models, with a reasonable compromise in accuracy. For example, Davies [24] studied the impact of excluding powder conduction, simplifying laser scan strategies and lumping multiple layers during thermal simulation. The simulation data is analyzed for accuracy by comparing it with the experimental temperatures recorded using thermocouples embedded inside the printed parts. It is reported that powder exclusion results in 4 times faster simulation. Furthermore, the layer-by-layer approach is reported to be 6 times faster than the model which simulates scan strategies. Lastly, lumping of 4 layers provided another 3 fold decrease in the computational time. It is reported that with increased layer lumping, the model captures an average evolution of the temperature field during the manufacturing process. Another study by Harrison [26] investigated the effect of using lumped layers as thick as 4,12 and 24 times the real layer thickness. The analysis was carried out for a 2D real-size part (height =54.7 mm, width =20.4 mm) and it was reported that lumping of 4 layers already results in a 20 times faster simulation compared to the one with no layer lumping. In both of these cases, although approximations provide significant computational gain, still the simulation times remain in the order of hours. For example, out of the two cases discussed above, the former reports that simulation with 4 layer lumping requires 1.33 h for parts with volume of 107 cm${}^{3}$ printed with 992 layers. For the latter 2D example, the model with lumping of 4 layers needed 4.6 h to complete.

In this paper, we aim to develop a model which could identify zones of local overheating and in order to assess manufacturability of designs and make quick design decisions, even faster computation times will be preferred. This is also motivated by the desire of integrating AM models within an optimization framework. Therefore, in this research, first a lumped layer-by-layer model is considered for simulating a real-size LPBF part and the temperature data is used to judge the heat accumulation tendencies of different design features. Details about this model are presented in Section 2. As highlighted in the previous paragraph, even with the simplifications of powder exclusion and lumped layer-by-layer deposition technique, the simulation time remains prohibitive for large scale implementations. Therefore in Section 3, six additional simplifications are presented. The first three have been partially discussed in the literature but an analysis of their impact on specifically overheating detection was never done before, which is discussed in Section 3.1. Next, three further simplifications are proposed in Section 3.2 which are the primary novel contributions of this paper. An analysis based on the analytical solution of the one-dimensional heat equation provides the motivation for these novel simplifications. Subsequently, Section 4 presents results obtained using the different simplifications and it is shown that the proposed simplifications provide adequate prediction of overheating regions while providing significant computation gains. Lastly, conclusions and future directions are discussed in Section 5.

## 2. Reference Thermal LPBF Process Model

In this section, a layer-by-layer part-scale thermal model is presented, intended for judging manufacturability of designs from the context of local overheating. It is based on the thermal model previously presented by Chiumenti et al. [27] for simulating material deposition in the laser material deposition (LMD) process. The same concept was later used for LPBF modeling in several publications [24,28,29]. The experimental validation of the model has been presented in Davies [24] where simulation results were compared with temperatures empirically recorded using in situ thermocouples placed inside the part during the LPBF process. It was shown that this modeling method can correctly capture the thermal history as obtained using the thermocouples. More importantly, it was observed by Neiva et al. [29] that the model correctly predicts the high temperatures measured near an overhanging feature. Therefore, it is deemed suitable for the intended purpose of identifying geometric features which cause local heat accumulation during the LPBF process. The model presented in this section acts as a reference for the simplified models presented in the later sections.

### 2.1. Model Description

**Figure 1:**
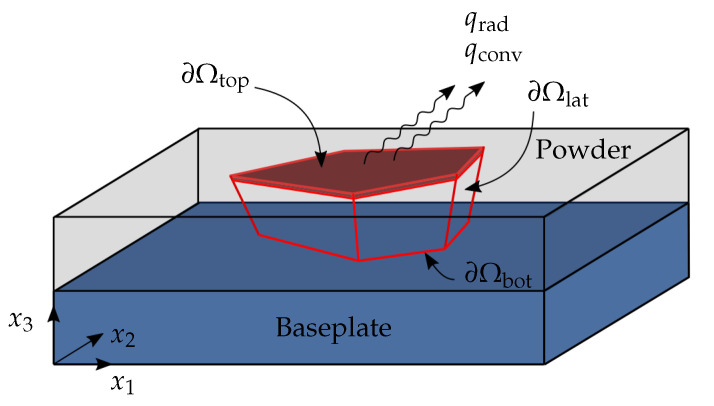
Schematic illustration of a body Ω being fabricated during the LPBF process. The topmost crimson colored region signifies the newly deposited layer. The body is attached to the baseplate at the bottom surface $\partial \Omega_{\mathrm{bot}}$ while a laser scans the top surface $\partial \Omega_{\mathrm{top}}$. The part-powder interface is denoted as the lateral surface $\partial \Omega_{\mathrm{lat}}$. Thermal losses due to convection and radiation are shown as $q_{\mathrm{conv}}$ and $q_{\mathrm{rad}}$, respectively.

A typical LPBF process includes layer-by-layer deposition of material on a thick baseplate. Figure 1 shows a schematic where an arbitrary 3D object is considered with volume Ω already deposited. The surfaces ∂Ω of the partly manufactured object are classified as top, lateral and bottom represented as $\partial \Omega_{\mathrm{top}}$, $\partial \Omega_{\mathrm{lat}}$ and $\partial \Omega_{\mathrm{bot}}$, respectively. The part remains submerged inside the powder bed while thermal energy provided through the newly deposited layer increases the part temperature in accordance with the heat equation given as

(1) $$\rho \left(T\right)c_{p}\left(T\right)\frac{\partial T}{\partial t}=\nabla \cdot \left(k\left(T\right)\nabla T\right)+Q_{v},$$

where *T* is temperature, *t* is time, $Q_{v}$ is a volumetric heat source and ρ, $c_{p}$, *k* are temperature-dependent density, specific heat and thermal conductivity, respectively.

Next, to formulate a boundary value problem (BVP), process-relevant boundary and initial conditions are specified. It is reported that the effective conductivity of powder layer is only 1% of bulk conductivity [30,31]. Hence, it is justified to assume that the heat transfer through powder is negligible as compared to conduction within the part. Therefore, lateral sides of the part, i.e., the part-powder interface, is assumed to be thermally insulated

(2) $$\frac{\partial T}{\partial x_{i}}n_{i}=0\;\mathrm{on}\;\partial \Omega_{\mathrm{lat}},$$

where $n_{i}$ are the components of the outward normal unit vector to $\partial \Omega_{\mathrm{lat}}$ and the repeated index indicates summation over *i*. The part remains bonded to the baseplate which is typically pre-heated and acts as a heat sink. This is taken into account by specifying a temperature boundary condition

(3) $$T=T_{0}\;\mathrm{on}\;\partial \Omega_{\mathrm{bot}},$$

where $T_{0}$ is the pre-heat temperature which is assumed to be constant. Next, heat losses through convection and radiation are considered at the top surface $\partial \Omega_{\mathrm{top}}$

(4) $$q_{\mathrm{conv}}=h_{\mathrm{conv}}\left(T-T_{a}\right)\;\mathrm{on}\;\partial \Omega_{\mathrm{top}},$$

where $h_{\mathrm{conv}}$ is the convective heat transfer coefficient and $T_{a}$ is the ambient temperature, assumed to be constant within the machine chamber. The radiative heat transfer boundary condition is given as

(5) $$q_{\mathrm{rad}}=\sigma \epsilon \left(T^{4}-{T_{a}}^{4}\right)\;\mathrm{on}\;\partial \Omega_{\mathrm{top}},$$

where σ is Stefan-Boltzmann constant and ϵ is emissivity of the radiating surface. Lastly, each newly added layer is assumed to be at an initial temperature of $T_{0}$.

It is important to note that during the process, melting and re-solidification of material also takes place. However, the influence of these phase transformations on resulting temperatures is not considered here. This is motivated by the fact that the energy absorbed during melting is released during re-solidification when the laser beam moves away from the melt zone. Hence, the net effect of phase transformations is negligible when considering part-scale temperatures [32]. As discussed in Section 1, lumped layer-by-layer material addition is considered here, which means that the uniform heat source $Q_{v}$ is simultaneously applied over the entire volume of the newly deposited lumped layer. Both of these simplifications, i.e., lumping and layer-by-layer material addition, have been motivated and applied in number of previous LPBF simulation studies [23,24,25,26,33]. Apart from obvious computational benefits, these choices are motivated by the fact that it has been shown by Davies [24] that a model with these simplifications is adequate for correctly capturing the part-scale thermal response.

### 2.2. Finite Element Analysis and Simulation Parameter Setting

To solve the boundary value problem (BVP) given by Equations (1)–(5), finite element analysis is used. The element birth-and-death method is implemented which is a common technique used to simulate the growing domain during the AM processes [20,26]. The implementation is done in CalculiX (a free and open source FE analysis code) [34] with a structured mesh where elements are aligned with the (lumped) layers. Eight-node linear cubic elements are used where mesh size is equal to the superlayer thickness which is assumed to be *S* = 500μm. This choice is motivated by the study conducted by Harrison [26] where temperature histories for different degree of layer lumping are compared. It is shown that the temperature response remains reasonably accurate for a superlayer thickness of 480μm for Ti-6Al-4V parts with typical LPBF machine parameters. Typically, layer thickness for the LPBF process varies from 20–80μm and in this study we use a representative value of 50μm for real LPBF layer thickness i.e., the superlayer is 10 times thicker than the real layer thickness. The newly deposited superlayer is subjected to a uniform volumetric heat source $Q_{v}$ [W/m${}^{3}$] for the heating time $t_{h}$. In accordance with Equation (3), the finite element nodes associated with the bottom surface act as heat sink, i.e., their temperature remains fixed at $T_{0}$. The heating step is followed by a cooling step in which the newly deposited layer can cool for an inter-layer time (ILT) $t_{d}$. The cycle of sequential heating and cooling repeats while the thermal history of all previous heating/cooling steps influence the simulation of the next step.

As mentioned earlier, the modeling principles presented by Davies [24] and Davies [28] are used here which provides a basis for setting simulation parameters $t_{h}$ and $Q_{v}$. The two key ideas presented by Davies [24] and Davies [28] are as follows:

- Heating time $t_{h}$ for a layer with area *A* equals the time that the laser would take for scanning that entire layer, i.e.,
  (6) $$t_{h}=\frac{A}{hv},$$
  where *h* is hatch spacing and *v* is laser scan velocity. Please note that by using this definition, $t_{h}$ is calculated for each super layer and it depends on the local part geometry through layer scan areas.
- It is ensured that the total deposited energy matches that of the actual process. Deposited energy per unit time is given by E=γP, where γ is the absorption coefficient and *P* is laser power. Using this principle, the volumetric heat source term can be calculated as
  (7) $$Q_{v}=\frac{\gamma P}{Al},$$
  where *l* is LPBF layer thickness.

Please note that the real LPBF layer thickness *l* which is different from the simulated superlayer thickness *S* is used for calculating $Q_{v}$ in Equation (7). This ensures that the input energy is automatically scaled when using thicker lumped layers. It is demonstrated by Davies [24] and Davies [28] that the described simulation scheme is capable of predicting the real physical temperatures recorded during the process as captured using the thermocouples located as close as 2.5 mm away from the topmost layer.

**Figure 2:**
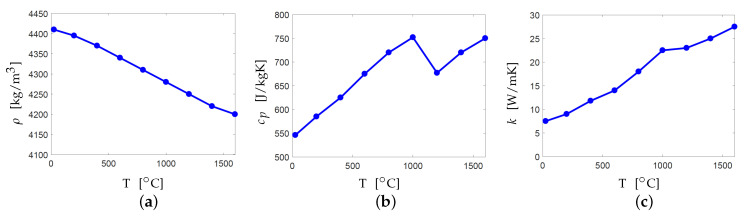
Variation of temperature-dependent bulk properties of Ti-6Al-4V. (**a**) Density (**b**) Specific heat and (**c**) Thermal conductivity [24].

Next, the ILT parameter used for cooling down between layer depositions needs to be specified. As mentioned, *S* = 500μm is used which is 10 times thicker than the real layer thickness. Hence, as suggested by Harrison [26], the ILT should also be scaled in order to compensate for the effect of large heat capacity of the superlayers. Hoelzle [23] used a method where ILT for a thick layer is adjusted such that thermal decay rates match with those of real-sized layers. However, the thermal decay rate varies at different locations within a part geometry which makes this estimation design dependent. Moreover, the ILT for the real size layers also depends on multiple factors such as layer area, scanning pattern, number of parts printed together inside the same chamber and recoater speed. Therefore, as per the linear scaling suggested by Harrison [26] and Malmelöv et al. [25], we choose to use an estimated value of 100 s which is 10 times the typical recoater time of 10 s and later we discuss the implications of this choice. The temperature-dependent properties of Ti-6Al-4V are taken from Davies [24] covering the range from room to fusion temperatures and shown in Figure 2. Finally, Table 1 lists the modeling parameters which are common to all the shown results.

### 2.3. Identification of Overheating Zones

**Figure 3:**
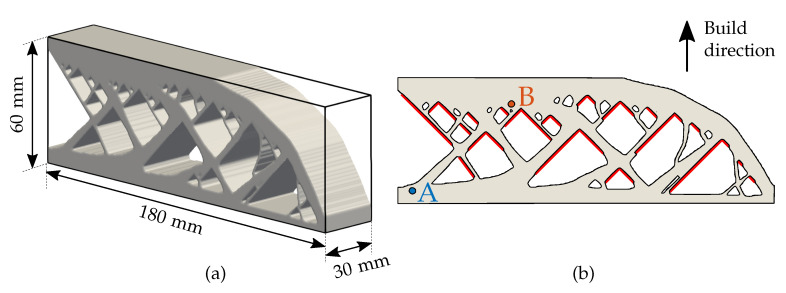
(**a**) The part geometry chosen for analysis with typical LPBF dimensions. (**b**) 2D cross section with overhangs marked with red lines.

Figure 3 illustrates the considered part geometry where dimensions and build direction are specified. The part dimensions are representative of a typical LPBF part. This particular design is chosen because of two key aspects. First, all overhanging features have the same overhang angle of $45^{\circ }$, marked with red lines in Figure 3b. In fact, the design is obtained by topology optimization method with overhang angle control as presented by Langelaar [35]. For our purpose, we choose this design to investigate whether the same overhang angle exhibits similar overheating behavior or not throughout the sample. Secondly, the design features do not change in the depth direction, i.e., the design is simply an extrusion of the 2D design shown in Figure 3b. This allows for convenient visualizations of temperatures fields as temperatures do not change in the direction of depth when considering layer-by-layer simulations.

**Figure 4:**
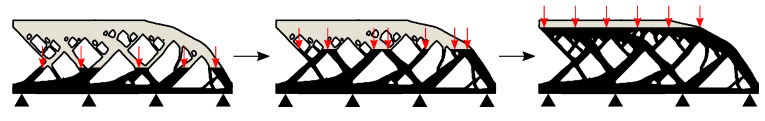
Three intermediate build instances with heat flux and sink boundary conditions represented by vertical arrows and triangles, respectively. The horizontal arrows indicate that thermal history of all previous heating/cooling steps are passed to the next simulation step.

**Figure 5:**
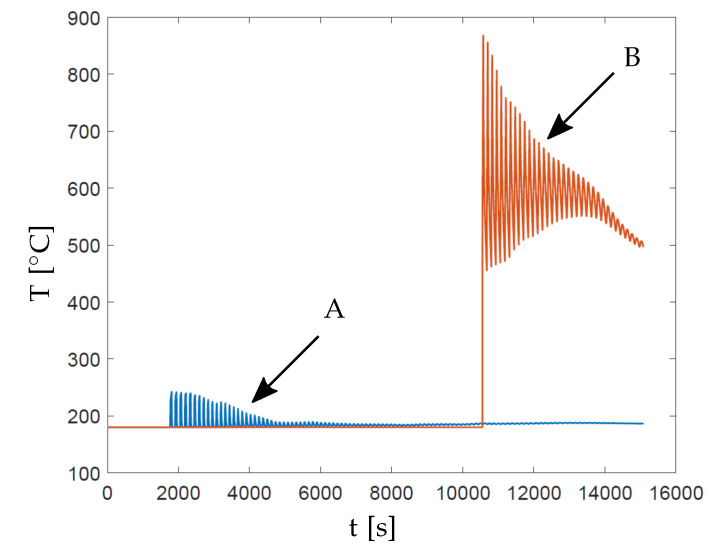
Variation of temperatures with respect to time for the node located at Points A and B in Figure 3b. Temperatures are calculated using the described transient thermal model of the LPBF process.

Figure 4 presents three intermediate build instances of this part where each new layer addition is followed by a heating and cooling step, where the peak temperatures occur at the end of the heating step. As explained earlier, heating time $t_{h}$ varies as per Equation (6), while ILT $t_{d}$ remains constant. Please note that in this model the thermal history of all previous heating/cooling steps is stored and influences the next step. Figure 5 shows typical variation of the temperature with respect to time for nodes located at two different locations marked as A and B in Figure 3b. First, note that the temperature first rises and reaches to a maximum when a layer is activated and then it rapidly drops during the inter-layer time. Next, the temperature rises again when the next layer is added. This phenomenon repeats until the final layer is deposited. Secondly, note that peak temperature attained at Point B is significantly higher than that at Point A. This is due to the geometric features in the vicinity of Point B, which do not allow for efficient heat evacuation, hence resulting in a higher peak temperature. Also, Point A is located closer to the baseplate facilitating quicker heat evacuation. This suggests that peak temperature value can be used as an indicator of overheating risk associated with a geometrical feature.

**Figure 6:**
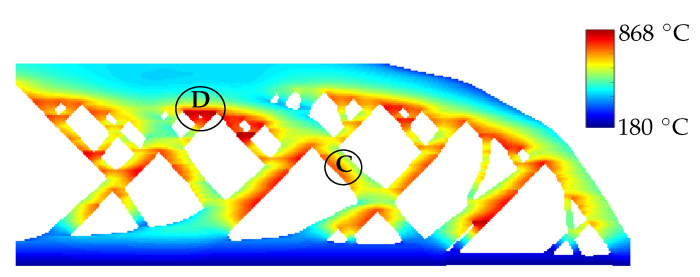
Contour plot for maximum temperature attained at each point of the part geometry during the entire build simulation using the layer-by-layer reference model. The region labelled as D shows higher maximum temperatures than that near the region C, while both regions are in the vicinity of a $45^{\circ }$ overhang. The temperature scale spans from initial temperature $T_{0}$ =180 ${}^{\circ }$C to the maximum value predicted by the simulation.

Figure 6 shows the peak temperatures attained for the entire domain during the build process. Please note that peak temperatures for different spatial locations may occur at different time instances during the simulation. It is evident that higher peak temperatures are exhibited near the overhangs, indicating local heat accumulation. Moreover, the funnel-shaped geometries in the region labelled as D in Figure 6 exhibit higher maximum temperatures than region labelled as C. This is in line with the experimental observations reported in Zimm. [17] and Patel et al. [18] where LBPF specimens exhibit local overheating in the vicinity of similar funnel-shaped features which act as a thermal bottleneck. This map of peak temperatures is used for judging design features for their heat accumulation behavior and referred to as the *hotspot map*. Moreover, although all features of the part have the same overhang angle of $45^{\circ }$, the thermal behavior in their vicinity is not similar. This implies that use of a purely geometric design rule can be insufficient for avoiding local overheating.

It is important to note that in the actual AM process, the temperatures rise till the melting point and then stabilize due to phase change with excess heat resulting in a larger melt-pool. Therefore, some LPBF simulation studies put an upper limit on the temperature to address this phenomenon, e.g., Childs [21]. However, for the reference model we choose not to use this concept and instead use the overshoot of temperature as an indicator of heat accumulation tendency of the neighboring geometry. It is still important to mention that the temperatures found using this model are representative values and are not exact in-process temperatures, due to the simplifications already introduced in the presented reference model. However, the relevance of the presented model for judging design manufacturability is demonstrated by the presented example where it is shown that peak temperatures detect overheating tendencies associated with geometric features.

Finally, the computational cost associated with the presented reference model remains significantly high due to the ever-growing analysis domain and the time integration to account for the transient nature of the problem. The model presented here is discretized using 2.16 million elements leading to 2.22 million nodal degrees of freedom (DOF) and the corresponding simulation time is 21 h 28 min 32 s. All computations reported in this paper are done on a HPC cluster node with 20 cores. The computational burden is still prohibitive for high-volume parts, interactive design iterations or integration with design optimization techniques. Therefore, to further reduce computational cost, additional physics-based simplifications are proposed in the next section.

## 3. Thermal Modeling Simplifications and Comparison Metrics

In total, six physics-based simplifications are presented in this section which are applied in addition to the simplifications already existing in the reference model detailed in Section 2. Section 3.1 discusses the influence of neglecting convective and radiative heat losses. Also, the effect of neglecting temperature-dependent properties is investigated. Although these simplifications have been applied in the literature, their impact in the context of detecting overheating was never studied. In Section 3.2, an analytical solution for one-dimensional heat equation is presented which serves as a basis for introducing three novel simplifications. With each simplification, a slightly different hotspot temperature field $T_{\mathrm{sim}}$ is obtained. Hence, to compare these with the reference hotspot temperature field $T_{\mathrm{ref}}$, three comparison metrics are defined in Section 3.3.

### 3.1. Influence of Neglecting Convective and Radiative Heat Losses

As discussed in Section 2.1 and described by Equations (4) and (5), heat is lost through convection and radiation from the topmost layer. However, the relative importance of heat losses through convection and radiation as compared to the conduction within the part is still debated in the literature. Some studies present arguments in favor of neglecting these losses [19,20,23], while others advocate for their inclusion in the thermal modeling of the AM processes [24,36]. A basic estimate can be made to quantify the thermal losses due to each mode of heat transfer. The convective loss near the melt zone can be estimated as

(8) $$q_{\mathrm{conv}}=h_{\mathrm{conv}}\left(T_{m}-T_{a}\right)\approx 2\times 10^{4}\;{W/m}^{2},$$

where $T_{m}$ is the melting point of Ti-6Al-4V taken as 1604 ${}^{\circ }$C and other parameters are given in Table 1. Similarly, radiative loss can be estimated as

(9) $$q_{\mathrm{rad}}=\sigma \epsilon \left(T_{m}^{4}-{T_{a}}^{4}\right)\approx 1.3\times 10^{5}\;{W/m}^{2}.$$

Next, in order to compare, the rate of energy transfer within the part due to conduction, $q_{\mathrm{cond}}$ can be estimated using a simplified version of Fourier’s law for one-dimensional heat flow:

(10) $$q_{\mathrm{cond}}\approx -k\frac{\Delta T}{\Delta x},$$

where ΔT is the temperature difference measured over a distance Δx. The melt zone is considered for making this estimation as the highest thermal gradients occur there. Consequently, conductivity at the melting point is considered in Equation (10). Due to extremely high thermal gradients and optical inaccessibility, it is extremely challenging to accurately record actual temperatures near the melt zone. Nevertheless, available data from the literature can be used to make an estimate. For example, empirical observations from Davies [28] reported a temperature difference ΔT=1200 ${}^{\circ }$C between the topmost point of the melt zone and a point located 2.5 mm below it, i.e., Δx=2.5 mm. Putting these values in Equation (10) gives |$q_{\mathrm{cond}}|=1.4\times 10^{7}\;{W/m}^{2}$. Please note that these are only rough estimates as heat fluxes continuously change with time-varying temperature fields. Moreover, this estimation of $q_{\mathrm{cond}}$ is an underestimation as even higher temperature gradients are observed in the vicinity of melt zone by melt-pool simulation studies [37]. Nevertheless, it can be observed from these estimates that indeed heat losses through convection and radiation are orders of magnitude lower near the melt zone than those by conduction, as argued by Paul et al. [19]. Also, radiation accounts for more heat loss than convection. The effect of neglecting one or both of these loss terms will be studied. In the remainder of this paper, simplification by exclusion of radiation is referred to as **S1** and that for excluding convection is termed as **S2**.

Another aspect that makes the PDE given by Equation (1) nonlinear, and consequently computationally expensive, is the temperature dependence of the thermal properties. Hence, another simplification (**S3**) is considered where along with exclusion of convection and radiation, constant thermal properties are considered. Ayas [32] and Yang et al. [38] performed a calibration study and showed that use of constant melting point properties are suitable when probing local temperatures near the heat deposition zone. Hence, in simplification **S3**, constant values of ρ=4200 kg/m${}^{3}$, $c_{p}=750$ J/kg K and k=27.5 W/m K are used. Results for detecting hotspots and computational gains achieved using these simplifications are reported in Section 4.

### 3.2. Novel Simplifications Motivated by One-Dimensional Heat Transfer Analysis

**Figure 7:**
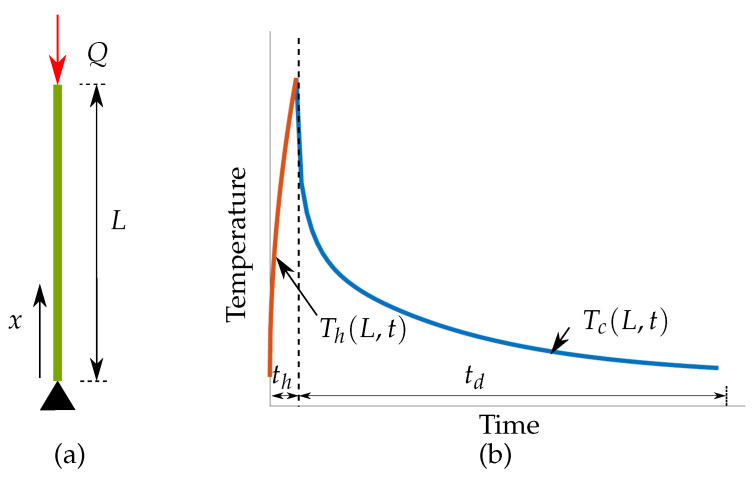
(**a**) one-dimensional domain with length *L* subjected to boundary conditions reminiscent to the reference model, i.e., heat flux *Q* acts at x=*L* while bottom temperature is fixed at T= $T_{s}$. (**b**) Thermal history of the topmost point (x=L) of the rod during a heating and cooling cycle.

To gain fundamental insights in the nature of transient heat transfer phenomena relevant to the LPBF process, a simplified case of one-dimensional heat transfer is considered and the analytical solution is presented for the boundary conditions analogous to the reference model. The detailed derivation is presented in Appendix A while the final solution and its implications are discussed here. A one-dimensional domain with length *L* is shown in Figure 7a. The heat equation given by Equation (1) can be simplified for one-dimensional heat transfer as

(11) $$\frac{1}{\alpha }\frac{\partial T}{\partial t}=\frac{\partial {}^{2}T}{\partial x^{2}},$$

where *x* is position and α is the thermal diffusivity given by $\alpha =k/\rho c_{p}$. Please note that the temperature dependence of thermal properties has been neglected for simplicity and constant values as given in Section 3.1 are used. First, a heating step is considered in which the topmost point x=*L* is subjected to a heat flux *Q* analogous to the volumetric heat source used in the reference model. Next, a heat sink condition is assumed at the bottom, i.e., x=0. To derive a more general solution, the sink temperature is specified as $T_{s}$ unlike the reference model where it was assumed same as the initial temperature $T_{0}$. These boundary conditions are given as

(12) $$-k\frac{\partial T}{\partial x}{|}_{x=L}=Q,\;\mathrm{and}$$

(13) $$T(0,t)=T_{s}\;\mathrm{for}\;t\geq 0.$$

The initial condition is

(14) $$T(x,0)=T_{0}\;\mathrm{for}\;x>0.$$

The rod is heated for a time $t_{h}$ followed by a cooling step in which *Q*=0. Using the method of separation of variables, the analytical solution is derived in Appendix A reads

(15) $$T_{h}\left(x,t\right)=T_{s}+T_{h}\left(L,\infty \right)\frac{x}{L}+2\sum_{n=1}^{\infty }\left(\frac{T_{0}-T_{s}}{\lambda_{n}}-\frac{T_{h}\left(L,\infty \right){\left(-1\right)}^{\left(n+1\right)}}{\lambda_{n}^{2}}\right)\sin\left(\lambda_{n}\frac{x}{L}\right)e^{-\frac{\lambda_{n}^{2}\alpha t}{L^{2}}},$$

where $T_{h}$ represents the temperature distribution during heating step and $T_{h}\left(L,\infty \right)=Q$L/k represents the steady-state temperature value at x=L while $\lambda_{n}=\left(2n-1\right)\pi /2$. Next, the rod is allowed to cool, i.e., the boundary condition given by Equation (12) becomes

(16) $$-k\frac{\partial T}{\partial x}{|}_{x=L}=0,$$

while the other boundary condition remains the same as given by Equation (13). For this case, the temperature distribution at the end of the heating step becomes the initial condition, i.e.,

(17) $$T\left(x,0\right)=T_{h}\left(x,t_{h}\right)\;\mathrm{for}\;x>0.$$

Again, the PDE given by Equation (11) is solved and the solution for the cooling regime is given as

(18) $$T_{c}\left(x,t\right)=T_{s}+2\sum_{n=1}^{\infty }\left(\frac{T_{h}\left(L,\infty \right){\left(-1\right)}^{\left(n+1\right)}}{\lambda_{n}^{2}}\left(1-e^{-\frac{\lambda_{n}^{2}\alpha t_{h}}{L^{2}}}\right)+\frac{T_{0}-T_{s}}{\lambda_{n}}e^{-\frac{\lambda_{n}^{2}\alpha t_{h}}{L^{2}}}\right)\sin\left(\lambda_{n}\frac{x}{L}\right)e^{-\frac{\lambda_{n}^{2}\alpha t}{L^{2}}},$$

where $T_{c}$ represents the temperature distribution during the cooling step. A visual depiction of the derived equations is given in Figure 7b where temperature variation for the topmost point (x=L) with respect to time is shown. Below, the derived solutions will be used to motivate three novel simplifications, aimed at further reduction of the computational burden associated with the reference model.

#### 3.2.1. Observation 1: Temporal Decoupling

First, we focus our attention on the magnitude of the temperature drop that occurs in the cooling phase between layer depositions, using the one-dimensional model. When this drop is sufficiently large, a decoupling in time of deposition steps can be considered in the process model. Recall from Section 2 that the peak temperatures at the end of the heating step are used to construct the hotspot map. Hence, the temperature difference between x=L and x=0 is considered. This difference during the cooling regime is normalized with the maximum temperature difference attained at the end of the heating step. The normalized temperature difference ${\hat{T}}_{c}\left(L,t\right)$ for the topmost point then reads

(19) $${\hat{T}}_{c}\left(L,t\right)=\frac{T_{c}\left(L,t\right)-T_{s}}{T_{h}\left(L,t_{h}\right)-T_{s}}=\frac{2\sum_{n=1}^{\infty }\frac{1}{\lambda_{n}^{2}}\left(e^{-\frac{\lambda_{n}^{2}\alpha t}{L^{2}}}-e^{-\frac{\lambda_{n}^{2}\alpha \left(t+t_{h}\right)}{L^{2}}}\right)}{1-2\sum_{n=1}^{\infty }\frac{1}{\lambda_{n}^{2}}e^{-\frac{\lambda_{n}^{2}\alpha t}{L^{2}}}}.$$

Please note that the sink temperature is assumed to be the same as the initial temperature in accordance to the boundary conditions used for defining the reference model, i.e., $T_{s}=T_{0}$. It can be deduced from Equation (19) that the cooling behavior depends on the duration of the heating stage $t_{h}$ and the characteristic time of the heat equation $\tau =L^{2}/\alpha$.

**Figure 8:**
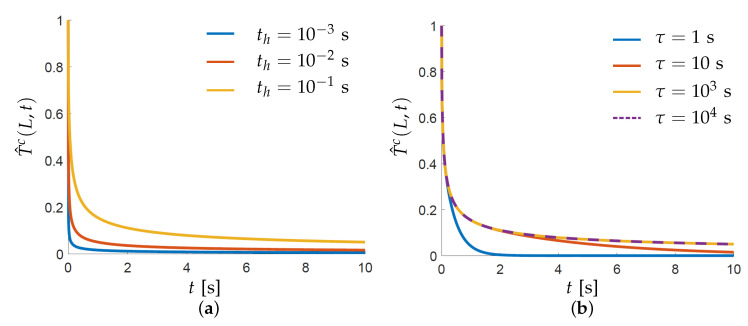
Variation of normalized cooling temperatures ${\hat{T}}^{c}$ with respect to time as given by Equation (19) for the considered one-dimensional rod illustrated in Figure 7a. (**a**) Plots for different values of heating time $t_{h}$ for $\tau =10^{4}$ s. (**b**) Plots for different values of characteristic time τ for $t_{h}=0.1$ s.

Figure 8a,b show the variation of ${\hat{T}}_{c}$ for a range of $t_{h}$ and τ values, respectively. These ranges are selected in the context of the LPBF process. For example, $t_{h}$ is typically very short in LPBF considering a typical scanning velocity v=1 m/s with a laser spot diameter of 100μm. Therefore, $t_{h}=10^{-3},10^{-2},10^{-1}$ s are selected to demonstrate the effect of heating time. Similarly, parts as high as 300 mm can be built in LPBF machines giving $\tau_{\max}\approx 10^{4}$ s, assuming α for Ti-6Al-4V. Hence, this value is used along with lower values to study the variation. Also, constant $\tau =10^{4}$ s and $t_{h}=0.01$ s are used for plotting graphs for varying $t_{h}$ and τ, i.e., Figure 8a,b, respectively. The infinite series in Equation (19) is converging and $n=10^{4}$ is found to be sufficient. Both graphs show that a slower cooling is observed as $t_{h}$ or τ increases. The graphs are shown for the first 10 s of cooling which is close to typical ILT values used in LPBF [11,23,26]. The crucial observation here is that in both graphs the topmost point cools down to approximately 10–20% of its highest temperature value in this time frame. This suggests that topmost point cools down to an estimated value of 150–300 ${}^{\circ }$C assuming $T_{m}$ as the highest temperature.

It is noteworthy that this finding is based on the simple one-dimensional model which assumes a constant conductivity throughout the domain. However, in the real three-dimensional setting, there could be local zones of lower conductivity near the topmost layer which would add to the heat accumulation and decrease the local cooling rate. As described in Section 1, examples of such features are acute overhangs and thin connections. Nevertheless, it is found that the findings based on this one-dimensional model are in agreement with the experimental observations reported by Davies [28] where thermocouples embedded inside an overhanging part recorded temperatures during the build. It is shown that the part temperatures remain in the range of 100–400 ${}^{\circ }$C at different locations below the topmost layer when the pre-heated baseplate temperature is at 100 ${}^{\circ }$C. This suggests that the one-dimensional insights can be extended to real parts. Another interesting observation made on the same data is that the recorded temperatures increase with the height at which thermocouples are placed. The effect of build height is also investigated by Hilgenberg [11] and slower cooling is reported for increasing part height. As a qualitative comparison, this is in accordance with cooling curves shown in Figure 8b, since $\tau \propto \;L^{2}$. It suggests that the effect of build height and thermal properties can be combined as characteristic time τ in order to study the cooling behavior. A more detailed quantitative comparison between τ and cooling data will better establish this correlation. However, it is deemed out of the scope of this paper.

**Figure 9:**
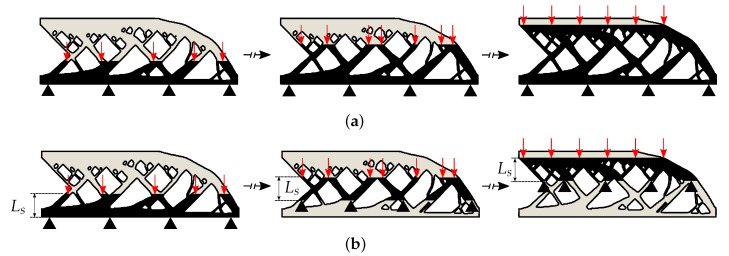
(**a**) Temporal decoupling: each new layer is assumed to be added at initial temperature $T_{0}$ and peak temperatures at the end of the heating step are used for preparing the hotspot map. This enables parallel simulation of all the layer additions providing computational gains. In this model, no data is shared between the simulations which is indicated by the horizontal broken arrows. (**b**) Spatial decoupling: only a relevant sub-geometry is considered for transient thermal analysis. This simplification is applied in addition to the temporal decoupling simplification introduced in (**a**).

The observations from the one-dimensional analytical model and experimental data from literature indicates that the ILT in the LPBF process allows for ample cooling between the layers. Moreover, it is also recommended as good manufacturing practice to design process such that appropriate cooling happens between two successive layers [11]. This suggests that the previously deposited layers do not significantly affect the peak temperatures recorded for the next layer. This enables decoupling the thermal history of different layers from each other for peak temperature prediction, which essentially means that each layer addition can be assumed to have an initial temperature equal to the baseplate temperature $T_{0}$. Peak temperatures attained this way would still capture the local overheating associated with the geometrical features of the part. A schematic representation of this idea is given in Figure 9a, where previous thermal history is not transferred to the next layer addition. There are two major computational benefits associated with this simplification. First, only simulation of the heating step suffices for capturing the first peak temperatures for each layer. Second, heating steps associated with every layer of the structure can be computed in parallel. The model which makes use of this simplification is referred to as the ‘temporally decoupled model’ and represented as **S4**. It is important to note that this model cannot capture the gradual heat accumulation that may occur over layer depositions, as information about the thermal history is lost. Nevertheless, it is found that features prone to local overheating can still be quickly and adequately identified when making use of this simplification. Recall that these features also contribute significantly to the gradual heat build-up that happens over the layers.

#### 3.2.2. Observation 2: Spatial Decoupling

Another useful observation is made by focusing on the relationship between the peak temperature at the end of the heating step $T^{h}\left(L,t_{h}\right)$ and domain size *L*. This implies substituting $t=t_{h}$, x=L and $T_{s}=T_{0}$ in Equation (15) which gives

(20) $$T_{h}\left(L,t_{h}\right)=T_{s}+T_{h}\left(L,\infty \right)\left(1-2\sum_{n=1}^{\infty }\frac{1}{\lambda_{n}^{2}}e^{-\frac{\lambda_{n}^{2}\alpha t_{h}}{L^{2}}}\right).$$

**Figure 10:**
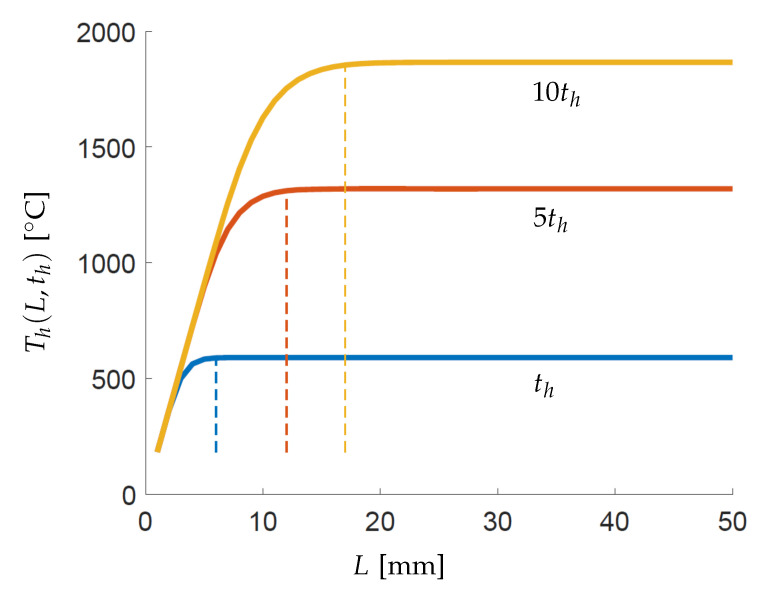
Variation of peak temperatures attained at the end of the heating step with respect to domain size *L* as described by Equation (20). Three graphs are shown for varying heating time $t_{h}$ and vertical lines are shown for respective Fo =0.3. First 10,000 terms of the infinite series given by Equation (20) are considered for plotting.

This relationship is pictorially presented in Figure 10 for three different heating times. It is evident that there exists a saturation domain length $L_{s}$ and increasing the domain size beyond this value has no effect on the peak temperatures. This phenomenon is characterized by the exponent term in Equation (20) which constitutes the Fourier number given as $Fo=\alpha t_{h}/L^{2}$. Please note that Fo is reducing along the horizontal axis in Figure 10 as *L* increases. It can be deduced from the graph that the saturation regime starts at Fo=0.3 which is marked by the vertical lines. Physically, it implies that for a given domain size *L*, thermal diffusivity α and heating time $t_{h}$, if Fo ≤0.3 then considering larger spatial domains will not influence the peak temperatures. It follows that the corresponding saturation length is $L_{s}$$=1.82\sqrt{\alpha t_{h}}$.

In the context of LPBF modeling, this observation implies that during the heating step, if the domain beyond $L_{s}$ is discarded from the analysis, peak temperatures will not be affected. Although derived using the one-dimensional model, the idea can be easily extended to higher dimensions where low conductivity regions influence the peak temperatures, only if they are present in the vicinity of topmost layer. Recall that temperature dependence is neglected, and the value of thermal properties estimated at the melting point are used for analytical derivation. However, since the highest value of α is achieved at the melting point, this choice gives an upper limit for the saturation length. Figure 9b shows an implementation of this idea, where the Dirichlet boundary condition given by Equation (3) is now applied at a distance $L_{s}$ below the heat source instead of at the baseplate. This simplification will be applied together with the previously introduced simplification of decoupling the layers in time. Hence, it is referred to as ‘spatially and temporally decoupled model’ and represented as **S5**. The reduced domain size enables further reduction of the computational cost.

#### 3.2.3. Observation 3: Steady-State Response for Detecting Overheating

It is well-known from the theory of heat conduction that lower conductivity regions inside a domain would influence the steady-state response of a thermal analysis [39]. Consequently, a steady-state response can also be used for detecting regions of lower conductivity within a given domain. The computation of steady-state responses is much faster than a transient analysis which makes it an attractive option for quickly detecting regions prone to overheating. In the context of the one-dimensional rod, substituting t=∞ in Equation (15), the steady-state temperature distribution along the length of the rod is found as

(21) $$T_{h}\left(x,\infty \right)=T_{s}+\frac{Qx}{k}.$$

**Figure 11:**
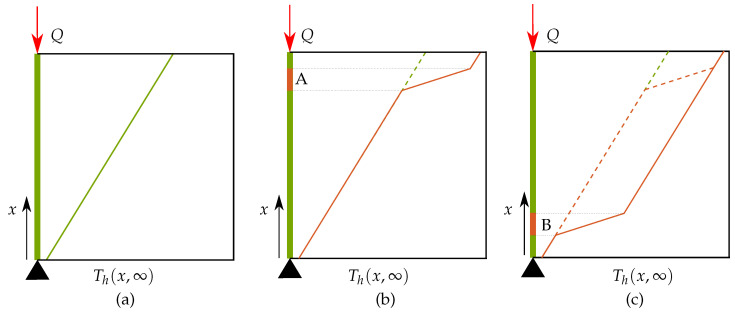
(**a**) Variation of steady-state temperature along the length of the domain as per Equation (21). (**b**) steady-state temperature with a patch of low conductivity located at A (**c**) steady-state temperature with a patch of low conductivity located at B.

This linear thermal profile is depicted in Figure 11a where *x* varies from 0 to *L* along the vertical axis while temperatures are plotted along the horizontal axis. Another case shown in Figure 11b considers that a subsection of the one-dimensional rod has lower thermal conductivity than the rest of the domain. This is shown by the orange patch at Location A. The steady-state temperature distribution for this case is computed and plotted, where a higher thermal gradient is observed in the patch of lower conductivity. Please note that also a higher steady-state temperature at x=L is found compared to the case without the patch, indicated by the dotted line. This signifies that the steady-state temperatures can also be used for identifying regions with lower conductivity which are prone to overheating. In three-dimensional setting, examples of such features are acute overhangs and thin connections which would create a local zone of lower conductivity.

However, the steady-state response should be cautiously used as it does not take into account the proximity of low conductivity regions to the topmost point where the heat flux is applied. This is illustrated in Figure 11c where the same low conductivity patch is now situated at Location B and still causes the same increase in the steady-state temperature at x=L. This implies that a low conductivity region situated far away from the heat deposition zone has the same effect on the top temperature when situated close to the topmost point, when analyzed using steady-state response. In context of the three-dimensional parts, this is unrealistic as heat transfer phenomenon is transient and only nearby regions of low conductivity (e.g., acute overhangs, thin connections) would influence the thermal behavior near the melt zone. Therefore, in order to rectify this inherent limitation of steady-state analysis, only the domain close to the topmost layer must be considered. Hence, this simplification is applied together with previously explained simplifications of temporal and spatial decoupling. In other words, instead of performing a transient analysis in the spatially and temporally decoupled model shown in Figure 9b, a computationally fast steady-state analysis is performed and the simplified model which involves this approximation is referred to as ‘steady-state model’ represented as **S6**.

### 3.3. Comparison Metrics

To assess the ability of each proposed model to detect hotspots in a part, three different comparison metrics are defined in order to capture different aspects associated with overheating detection. Recall that reference and simplified temperature fields are referred to as $T_{\mathrm{ref}}$ and $T_{\mathrm{sim}}$, respectively. The first metric is defined as the percentage error between the maximum temperatures recorded for both fields,

(22) $$\delta =\frac{\max\left(T_{\mathrm{sim}}\right)-\max\left(T_{\mathrm{ref}}\right)}{\max(T_{\mathrm{ref}})}\times 100\%.$$

This quantity compares the intensity of worst overheating as predicted by the reference and the simplified models. It indicates how much the corresponding simplification under/over-predicts as compared to the reference case. A positive δ signifies over-prediction and vice versa. Please note that this quantity compares the absolute maxima between two temperature fields without requiring that these maxima occur at the same spatial point. Therefore, to assess the spatial similarity between two temperature fields, the Jaccard index is used. This is a measure commonly used in the field of image recognition for quantifying similarity between two images and is defined as the ratio of intersection and union between the two images [40]. For our case, first T is normalized using max(T), i.e., $\hat{T}=T/\max\left(T\right)$, where $\hat{T}$ indicates the normalized temperature field. Next, the Jaccard index is defined as

(23) $$J=\frac{\sum_{i=1}^{n}\min\left({\hat{T}}_{\mathrm{ref}}^{\left(i\right)},{\hat{T}}_{\mathrm{sim}}^{\left(i\right)}\right)}{\sum_{i=1}^{n}\max\left({\hat{T}}_{\mathrm{ref}}^{\left(i\right)},{\hat{T}}_{\mathrm{sim}}^{\left(i\right)}\right)}\times 100\;\%,$$

where ${\hat{T}}_{\mathrm{ref}}^{\left(i\right)}$, ${\hat{T}}_{\mathrm{sim}}^{\left(i\right)}$ represent normalized temperature values for node *i* for the reference and the simplified fields, respectively and *n* is the total number of nodes in the finite element analysis. A high Jaccard index implies that the overall temperature distributions over the part are highly similar.

The third metric is defined for a qualitative comparison of two hotspot fields. A critical zone identification (CZI) map is defined which highlights the worst overheated zones while suppressing the cooler, less critical, regions in a given geometry. This allows for judging simplifications based on their capability of detecting the correct worst overheating locations while the actual temperature predictions might be significantly off. It is defined as the contour map of the normalized temperature field with four contour levels, here chosen at 50%,70%, 80% and 90% of the maximum temperature in the field. Using this, different design features can be identified from least to most critical in terms of overheating.

Finally, in order to quantify the degree of computational gain achieved by a simplification, a gain factor is defined as

(24) $$\eta =\frac{t_{\mathrm{ref}}^{c}}{t_{\mathrm{sim}}^{c}},$$

where $t_{\mathrm{ref}}^{c}$ and $t_{\mathrm{sim}}^{c}$ are the wall-clock times for completing the reference and simplified analysis, respectively.

## 4. Numerical Results and Discussion

**Figure 12:**
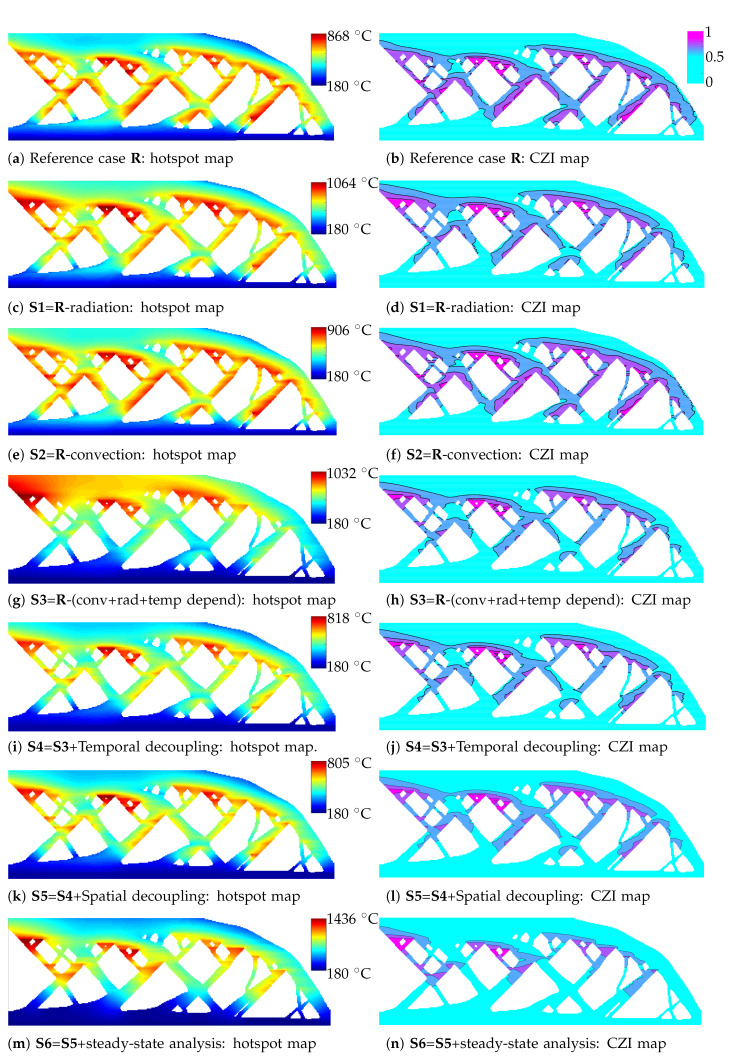
Peak temperature plots, i.e., the hotspot maps for different simplifications are presented in (**a**,**c**,**e**,**g**,**i**,**k**) and (**m**) while the CZI maps for different simplifications are presented in (**b**,**d**,**f**,**h**,**j**,**l**,**n**). Sub-captions are provided to specify the respective simplifications.

To illustrate the validity of the proposed simplifications and discuss the associated implications, hotspot maps for the part shown in Figure 3 are generated using simplified models. For ease of visual comparison, all hotspot fields are presented in Figure 12 along with corresponding CZI maps. Simulation times and calculated comparison metrics are listed in Table 2 while the default simulation parameters are used from Table 1. All computations are performed using 20 cores on a HPC cluster. Discussion about each simplification is presented in the same order in which simplifications were introduced in Section 3.

### 4.1. Hotspot Map without Considering Convective/Radiative Heat Losses

To compare the relative importance of convection and radiation heat losses, two hotspot maps are prepared. One by excluding radiation, shown in Figure 12c and other by excluding convection, shown in Figure 12e. All other simulation parameters remain the same as in the case of the reference model, depicted in Figure 12a. The first observation is that neglecting convection or radiation leads to a conservative estimation of overheating zones. This is due to the fact that both convection and radiation contribute to the thermal losses and hence lead to higher simulation temperatures, when excluded. Also, as estimated, exclusion of radiation has a greater impact than exclusion of convection. It is manifested by the fact that the maximum temperature attained by excluding radiation is 22.4% higher than that obtained with the reference model, i.e., δ(S1)=22.4%. On the other hand, δ(S2)=4.2% when convection is excluded from the analysis. Moreover, when looking at the overall temperature distribution over the entire domain, the hotspot map obtained considering radiation while neglecting convection is very similar to that obtained using reference model. This is supported by the Jaccard index J(S2)=96.4%. Please note that even with exclusion of convection or radiation, the hotspot map still correctly predicts the funnel-shaped geometries as the worst overheating features. This is also clear from the CZI maps shown in Figure 12b,d,f where the same features are identified as critical. Lastly, excluding radiation and convection provides marginal computational gains of η(S1)=1.06 and η(S1)=1.03, respectively. The radiation boundary condition is nonlinear and hence its exclusion provides a slightly higher computational gain.

### 4.2. Hotspot Map Without Considering Temperature-Dependent Properties

The influence of neglecting temperature dependence of thermal properties is studied next. The hotspot map presented in Figure 12g considers this simplification in addition to the simplifications of excluding convection and radiation. The thermal properties as listed in Section 3.1 are used for preparing this map. An interesting observation here is that a significant heat accumulation is observed between the layers. This is indicated by the temperature gradient that is seen in the build direction where the topmost layers exhibit significantly higher peak temperatures than the reference model. Due to this, the overall distribution of the peak temperatures over the domain is considerably different from those found using the reference model. This difference is quantified by J(S3)=75.4%. Moreover, the maximum temperature is overestimated by δ(S3)=19%. Nevertheless, the funnel-shaped geometries are still detected as the worst overheating zones, as shown in the CZI map presented in Figure 12h. The computational gain factor is found to be η(S3)=2.1.

The observations made using these simplifications can be used for making appropriate modeling choices when investigating different aspects of the LPBF process. It is shown here that the exclusion of convection, radiation and temperature dependence halves the simulation time while correctly detecting design features responsible for severe local overheating. However, if the aim of the simulation is to study the effect of gradual heat accumulation during the build, for example as conducted by Jamshidinia and Kovacevic [41], then using these simplifications might lead to overly conservative fields and, thus, leading to false positives. This will become more critical in the case of tall parts with high heat capacity and/or processes with short ILT. Therefore, suitable modeling choices should be made.

### 4.3. Hotspot Map with Temporal Decoupling

Inspired by the observations from the analytical model, the simplification of decoupling layer addition is studied. The hotspot map in Figure 12i shows the peak temperatures obtained at the end of the heating step. The computations associated with each intermediate layer addition are carried out in parallel and the hotspot map is prepared as a post-simulation step. As discussed in Section 3, this simplification assumes constant initial temperature $T_{0}$ for each new layer addition. In other words, it assumes that the part cools down to $T_{0}$ between successive layer additions. Consequently, this simplification cannot capture the effect of gradual heat accumulation which builds up over the layers. In fact, this effect is opposite to the preceding case (**S3**) where gradual heat build-up was overestimated. This is also evident by the fact that peak temperatures are underestimated here, as quantified by δ(S4)=−5.8%. Nevertheless, the hotspot map prepared using this simplification yields result very similar to the reference case. This is evident by comparing the CZI maps shown in Figure 12b,j which highlight same set of design features. This is also quantified by high similarity between the two temperature fields with J(S4)=89.9%. Lastly, a computational gain factor η(S4)=85.2 is achieved where thermal analysis takes only 15 m 7 s while the difference between peak temperature values δ(S4) remains less than 6%. Recall that reduction in wall-clock time is attributed to the parallel simulation of each new layer addition and omission of the cooling step simulation between the layers. As mentioned, this model cannot capture the gradual heat accumulated during the build which is inversely proportional to the ILT [11], and similar precautions apply as mentioned for **S3**.

### 4.4. Hotspot Map with Spatial Decoupling

As per the observation discussed in Section 3.2.2, the simplification of considering only the thermally relevant domain during the heat addition step is applied and the resulting hotspot map is presented in Figure 12k. Recall that this simplification is applied in addition to the simplification of decoupling in time, as shown in Figure 9b. The domain size for each new layer addition is calculated using the concept of saturation length presented in Section 3.2.2. The resulting hotspot map is found to be very similar to the one found using the concept of temporal decoupling (**S4**). This signifies that very small additional errors are introduced when considering local domains instead of the full geometry. Reduction of the size of simulation domain provides a further improvement in computational gain factor, which reaches 144.2.

### 4.5. Hotspot Map with Steady-State Analysis

Next, use of computationally fast steady-state analysis instead of the transient response is studied. The hotspot map shown in Figure 12m shows the obtained hotspot map with steady-state peak temperatures. As expected, the temperature values are significantly higher than those found using the transient analysis. Moreover, the overall temperature distribution is also considerably different. These are quantified by δ(S6)=65.3% and J(S6)=74.8%. Nevertheless, the hotspot map found using steady-state analysis also identifies the correct critical zones of overheating, as observed in the CZI map presented in Figure 12n. The analysis is completed in only 2 min 9 s with a computational gain factor of 600.

### 4.6. Comparative Analysis

**Figure 13:**
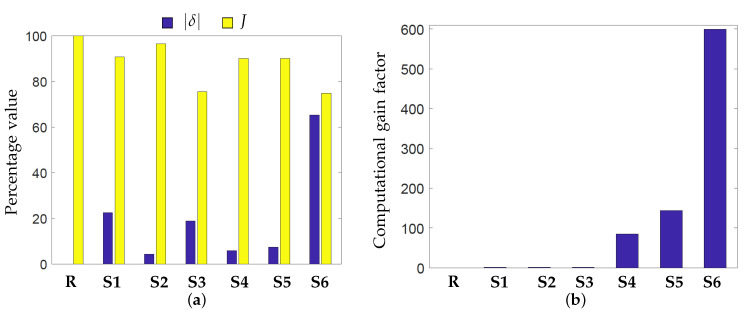
(**a**) Comparison metrics δ and *J* for judging the accuracy of the simplifications from the context of detecting heat accumulation. Low δ and high *J* implies higher conformance with the reference case. (**b**) Computational gain factors η for the simplifications.

A pictorial representation of maximum percentage error δ and Jaccard index *J* obtained using different simplifications is given in Figure 13a and computational gain factors are plotted in Figure 13b. Please note that δ is a measure of difference between two fields while *J* is a measure of similarity. Therefore, the simplifications which yield low δ and high *J* signify higher conformance with the reference hotspot map. Upon comparison, it becomes clear from Figure 13a that the analysis which includes radiation (**S2**) is more accurate than the one which excludes it (**S1**). Also, the analysis with decoupled layers (**S4**) and local domain (**S5**) are almost the same in terms of accuracy. Figure 13b highlights the considerable computational gains provided by the novel simplifications proposed in this paper. Please note that these gains are achieved in part due to the parallel processing, which becomes possible due to the proposed simplifications. The total processing CPU times are also reported in Table 2 which presents the total computation time used by all the processors. This also implies that the wall-clock time will directly depend on the number of processors used. Please note that even without using parallel processing, the novel simplifications provide significant gains where the steady-state model is still 15 times faster than the reference model when CPU times are compared. To conclude, a summary of disadvantages or risk associated with each simplification is presented in Table 3.

## 5. Conclusions and Future Work

In this paper, first an established thermal model is used to predict the overheating behavior in an LPBF part. A representative part with a 162 cm${}^{3}$ deposition volume is considered which is simulated using 2.22 million DOFs. It is revealed that using purely geometric design guidelines might not be sufficient for avoiding overheating. It is shown that even with the simplifications of layer lumping and simultaneous simulation of entire layer deposition, the computational time is still prohibitive for quick manufacturability assessment needed for efficient design modifications, process-parameter tuning and optimizations. Hence, a total of six further simplifications are investigated, ranging from omission of radiation and convection and use of constant material properties, to novel simplifications involving temporal and spatial decoupling and ultimately the use of localized steady-state responses. It is shown that in particular the three novel simplifications provide very high computational gains. The results from the simplifications of temporal and spatial decoupling show that even after omitting the simulation of the cooling step and reducing the computational domain size, these simplifications can still provide crucial information about design features and their thermal behavior during the LPBF process. Using these simplifications, the correct locations prone to overheating can be identified and error in maximum temperature prediction is less than 10% when compared with the reference case. Moreover, the localized steady-state response is shown to provide accurate qualitative information about the location of problematic features while it is 600 times faster than the reference model. Note that this computational gain directly depends on the number of processors used.

The high computational gains achieved using simplified models makes them especially suitable for design optimization problems where several hundred design evaluations might be needed for finding the optimized design. For example, integration of simplified models with topology optimization techniques holds the promise to deliver highly efficient designs which are also robust from the context of overheating. Typically, design optimization methods make use of gradient information and it has been shown by Van Keulen et al. [42] that gradient computation for a transient analysis is computationally much more expensive as compared to a steady-state analysis. Consequently, the steady-state model which correctly identifies overheating zones is a perfect candidate for integration with topology optimization, which is seen as the immediate next step.

The layer-by-layer model used in this research cannot capture the influence of laser scanning vectors on local overheating. Therefore, development of higher fidelity models which simulate laser movement and analysis of the generated thermal history for identification of local overheating is seen as a future step. It is expected that the introduced simplifications of temporal and spatial decoupling would remain valid for such higher fidelity models. However, thorough investigation of their validity would still be needed and seen as a possible research step. The thermal history also dictates the development of residual stresses which cause deformations and even part failures. Therefore, the possibility of extending simplified thermal models for capturing mechanical aspects is a promising research direction. Another avenue of future research is to experimentally validate the simplified models. For this purpose, a study is underway where the beam design investigated in this paper is manufactured and a metallographic study is being performed. The specimen will be cut and examined near the funnel shapes which are identified as critical zones by the simplified models.

## Figures and Tables

**Table 1:** List of modeling parameters used for thermal analysis of LPBF process.

*P* [W]	γ	*v* [ms${}^{-1}$]	*h* [mm]	*l* [mm]	*S* [mm]	$T_{0}$ [${}^{\circ }$C]	$T_{a}$ [${}^{\circ }$C]	$h_{\mathrm{conv}}$ [Wm${}^{-2}$K${}^{-1}$]	ϵ
200	0.45	1	0.14	0.05	0.5	180	25	10	0.35

**Table 2:** Comparison of maximum percentage error δ, Jaccard index *J*, simulation times and computational gains for all simplified models. All computations are performed using 20 cores on a HPC cluster.

Model Description	Wall-Clock Time	CPU Time	δ	*J*	η
**R**: Reference case	21 h 28 min 32 s	20 h 52 min 38 s	0	100	1
**S1**: **R**-radiation	20 h 11 min 6 s	19 h 35 min 6 s	22.4	90.6	1.06
**S2**:**R**-convection	20 h 39 min 27 s	20 h 3 min 30 s	4.2	96.4	1.03
**S3**: **R**-(rad, conv, temp depend)	15 h 3 min 29 s	14 h 48 min 16 s	18.8	75.4	1.7
**S4**: **S3**+Temporally decoupled	15 min 7 s	13 h 20 min 27 s	−5.8	89.9	85.2
**S5**: **S4**+Spatially decoupled	8 min 6 s	7 h 18 min 7 s	−7.2	89.9	144.2
**S6**: **S5**+Steady-state model	2 min 9 s	1 h 25 min 29 s	65.3	74.8	599.3

**Table 3:** A summary of disadvantages associated with each simplification.

Model Description	Disadvantage
**S1**: **R**-radiation	Conservative prediction, risk of false positives
**S2**:**R**-convection	Conservative prediction, risk of false positives
**S3**: **R**-(radiation, convection, temp. dependent)	Conservative prediction, risk of false positives
**S4**: **S3**+Temporally decoupled	Thermal history lost, cannot capture gradual heat build-up
**S5**: **S4**+Spatially decoupled	Thermal history lost, cannot capture gradual heat build-up
**S6**: **S5**+Steady-state model	Qualitative indication only

## Appendix A. Derivation of Analytical Solution for One-Dimensional Heat Equation

We consider the one-dimensional heat equation given by Equation (11). The boundary conditions for the heating regime and the initial condition are given by Equations (12)–(14), respectively. Note that these are non-homogeneous, while the method of separation of variables can be applied only for homogeneous BCs. Therefore, following transformations are introduced

(A1) $$\tilde{x}=\frac{x}{L},$$

(A2) $$\tilde{t}=\frac{t}{\tau },$$

(A3) $$\tilde{T}=T-T_{s}-T_{\mathrm{ss}}\tilde{x},$$

where $\tau =L^{2}/\alpha$ and $T_{\mathrm{ss}}=\left(QL\right)/k$. Using Equations (A1)–(A3) the heat equation is transformed as

(A4) $$\frac{\partial \tilde{T}}{\partial \tilde{t}}=\frac{\partial^{2}\tilde{T}}{\partial {\tilde{x}}^{2}},$$

while the flux BC becomes

(A5) $$\frac{\partial \tilde{T}}{\partial \tilde{x}}{|}_{\tilde{x}=1}=0.$$

The other boundary condition i.e., $T=T_{s}$ at x=0 and the initial condition given by Equation (14) also gets modified as

(A6) $$\tilde{T}\left(0,\tilde{t}\right)=0\;\mathrm{for}\;\hat{t}\geq 0,$$

(A7) $$\tilde{T}\left(\tilde{x},0\right)=T_{0}-T_{s}-T_{\mathrm{ss}}\tilde{x}=p\left(\tilde{x}\right)\;\mathrm{for}\;\hat{x}>0.$$

The boundary conditions are now homogeneous. Hence, method of separation of variable can be used. Using which, $\tilde{T}$ is assumed to be a combination of two separate functions, i.e.,

(A8) $$\tilde{T}=X\left(\tilde{x}\right)\psi \left(\tilde{t}\right),$$

where *X* and ψ are functions of $\tilde{x}$ and $\tilde{t}$ alone, respectively. Putting Equation (A8) in Equation (A4), the general solution can be worked out as

(A9) $$\tilde{T}\left(\tilde{x},\tilde{t}\right)=\left(A\sin\left(\lambda \tilde{x}\right)+B\cos\left(\lambda \tilde{x}\right)\right)\left(Ce^{-\lambda^{2}\tilde{t}}\right),$$

where A,B and *C* are constants. Next, we use the boundary conditions for determining the values for these constants. Using boundary condition described by Equation (A6) and ignoring trivial solutions, it can be worked out that B=0. Putting this in Equation (A9), we get

(A10) $$\tilde{T}\left(\tilde{x},\tilde{t}\right)=D\sin\left(\lambda \tilde{x}\right)e^{-\lambda^{2}\tilde{t}},$$

where D=AC. Now, we use the other boundary condition described by Equation (A5) which yields

(A11) $$\frac{\partial \tilde{T}}{\partial \tilde{x}}{|}_{\tilde{x}=1}=D\lambda \cos\left(\lambda \right)e^{-\lambda^{2}\tilde{t}}=0,$$

which gives

(A12) $$\lambda_{n}=\frac{2n-1}{2}\pi \;\mathrm{for}\;n=1,2,3....$$

Now we apply principle of superposition, which means that if the solution holds true for one λ, then it must be true for a linear combination of all $\lambda_{n}$. Hence,

(A13) $$\tilde{T}\left(\tilde{x},\tilde{t}\right)=\sum_{n=1}^{\infty }D_{n}\sin\left(\lambda_{n}\tilde{x}\right)e^{-\lambda_{n}^{2}\tilde{t}},$$

combines the solutions for all possible λ values. Finally, we utilize the initial condition given by Equation (A7) for determining the constant $D_{n}$ as

(A14) $$\tilde{T}\left(\tilde{x},0\right)=\sum_{n=1}^{\infty }D_{n}\sin\left(\lambda_{n}\tilde{x}\right),$$

which should be equal to the temperature distribution given by Equation (A7). This gives

(A15) $$p\left(\tilde{x}\right)=\sum_{n=1}^{\infty }D_{n}\sin\left(\lambda_{n}\tilde{x}\right).$$

For determination of $D_{n}$, orthogonality property of the function $\sin(\lambda_{n}\tilde{x})$ is utilized. This gives

(A16) $$D_{n}=\frac{\int_{0}^{1}p\left(\tilde{x}\right)\sin\left(\lambda_{n}\tilde{x}\right)d\tilde{x}}{\int_{0}^{1}\sin^{2}\left(\lambda_{n}\tilde{x}\right)d\tilde{x}},$$

or

(A17) $$D_{n}=2\int_{0}^{1}p\left(\tilde{x}\right)\sin\left(\lambda_{n}\tilde{x}\right))d\tilde{x},$$

as denominator of Equation (A16) becomes 1/2. Now, putting the value of $p(\tilde{x})$ from Equation (A7), we get

(A18) $$D_{n}=2\int_{0}^{1}(T_{0}-T_{\mathrm{ss}}\tilde{x}-T_{s})\sin\left(\lambda_{n}\tilde{x}\right)d\tilde{x}.$$

Integration yields

(A19) $$D_{n}=\frac{2(T_{0}-T_{s})}{\lambda_{n}}-\frac{2T_{\mathrm{ss}}{\left(-1\right)}^{\left(n+1\right)}}{\lambda_{n}^{2}}.$$

Finally, we substitute $D_{n}$ from Equation (A19) into Equation (A13) and subsequently use Equation (A3), to get temperature distribution during the heating regime as

(A20) $$T_{h}\left(x,t\right)=T_{s}+T_{\mathrm{ss}}\frac{x}{L}+2\sum_{n=1}^{\infty }\left(\frac{\left(T_{0}-T_{s}\right)}{\lambda_{n}}-\frac{T_{\mathrm{ss}}{\left(-1\right)}^{\left(n+1\right)}}{\lambda_{n}^{2}}\right)\sin\left(\lambda_{n}\frac{x}{L}\right)e^{-\frac{\lambda_{n}^{2}\alpha t}{L^{2}}}.$$

Note that $T_{\mathrm{ss}}=\left(QL\right)/k$ is same as the steady-state temperature at x=L for the heating step, i.e., $T_{\mathrm{ss}}=T_{h}\left(L,\infty \right)$.

The derivation for cooling regime remains exactly the same except the initial condition is now prescribed by the temperature distribution at the end of the heating step, i.e.,

(A21) $$\tilde{T}\left(\tilde{x},0\right)=p_{h}\left(\tilde{x}\right)=T_{h}\left(\tilde{x},{\tilde{t}}_{h}\right)\;\mathrm{for}\;\hat{x}>0.$$

Therefore, replacing $p(\tilde{x})$ by $p_{h}\left(\tilde{x}\right)$ in Equation (A17), integrating and using Equation (A3) leads to the solution for cooling regime as

(A22) $$T_{c}\left(x,t\right)=T_{s}+2\sum_{n=1}^{\infty }\left(\frac{T_{\mathrm{ss}}{\left(-1\right)}^{\left(n+1\right)}}{\lambda_{n}^{2}}\left(1-e^{-\frac{\lambda_{n}^{2}\alpha t_{h}}{L^{2}}}\right)+\frac{\left(T_{0}-T_{s}\right)}{\lambda_{n}}e^{-\frac{\lambda_{n}^{2}\alpha t_{h}}{L^{2}}}\right)\sin\left(\lambda_{n}\frac{x}{L}\right)e^{-\frac{\lambda_{n}^{2}\alpha t}{L^{2}}}.$$
